# Element Changes Occurring in Brain Point at the White Matter Abnormalities in Rats Exposed to the Ketogenic Diet During Prenatal Life

**DOI:** 10.1021/acschemneuro.4c00283

**Published:** 2024-10-23

**Authors:** Marzena Rugieł, Zuzanna Setkowicz, Mateusz Czyzycki, Rolf Simon, Tilo Baumbach, Joanna Chwiej

**Affiliations:** 1 Faculty of Physics and Applied Computer Science, AGH University of Krakow, Al. Mickiewicza 30, Krakow 30-059, Poland; 2 Institute of Zoology and Biomedical Research, Jagiellonian University, Gronostajowa 9, Krakow 30-387, Poland; 3 Institute for Photon Science and Synchrotron Radiation, Karlsruhe Institute of Technology, Hermann-von-Helmholtz-Platz 1, Eggenstein-Leopoldshafen D-76344, Germany; 4 Laboratory for Applications of Synchrotron Radiation, Karlsruhe Institute of Technology, Kaiserstr. 12, Karlsruhe D-76131, Germany

**Keywords:** ketogenic diet, synchrotron X-ray fluorescence microscopy, multielement analysis, prenatal exposure, animal models, brain development

## Abstract

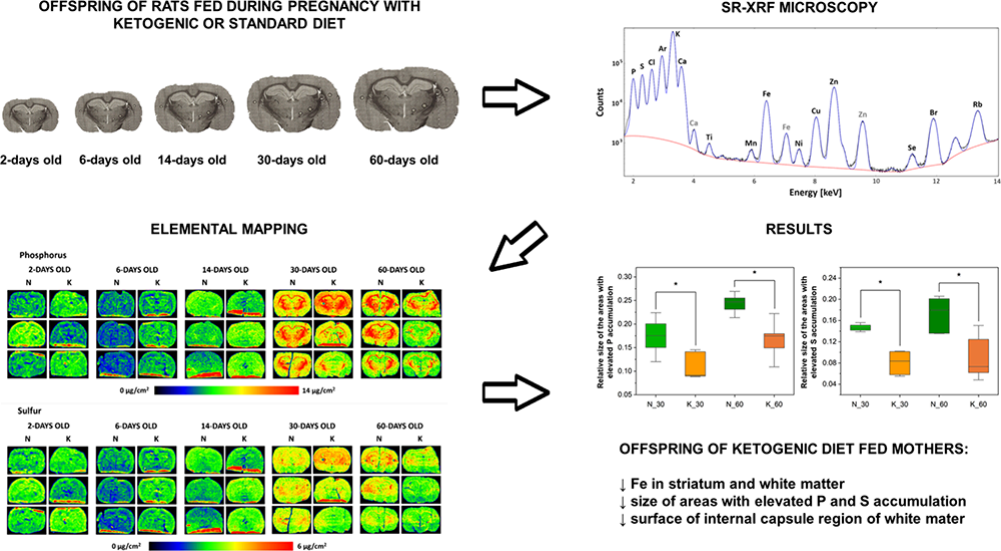

A large number of clinical studies demonstrate that the ketogenic diet (KD) may be an effective approach to the reduction of epileptic seizures in children and adults. Such dietary therapy could also help pregnant women with epilepsy, especially since most antiseizure drugs have teratogenic action. However, there is a lack of medical data, considering the safety of using KD during gestation for the progeny. Therefore, we examined the influence of KD used prenatally in rats on the elemental composition of the selected brain regions in their offspring. For this purpose, synchrotron radiation-induced X-ray fluorescence (SR-XRF) microscopy was utilized, and elements such as P, S, K, Ca, Fe, and Zn were determined. Moreover, to verify whether the possible effects of KD are temporary or long-term, different stages of animal postnatal development were taken into account in our experiment. The obtained results confirmed the great applicability of SR-XRF microscopy to track the element changes occurring in the brain during postnatal development as well as those induced by prenatal exposure to the high-fat diet. The topographic analysis of the brains taken from offspring of mothers fed with KD during pregnancy and appropriate control individuals showed a potential influence of such dietary treatment on the brain levels of elements such as P and S. In the oldest progeny, a significant reduction of the surface of brain areas characterized by an increased P and S content, which histologically/morphologically correspond to white matter structures, was noticed. In turn, quantitative elemental analysis showed significantly decreased levels of Fe in the striatum and white matter of 30-day-old rats exposed prenatally to KD. This effect was temporary and was not noticed in adult animals. The observed abnormalities may be related to the changes in the accumulation of sphingomyelin and sulfatides and may testify about disturbances in the structure and integrity of the myelin, present in the white matter.

## Introduction

The ketogenic diet (KD) is a dietary therapy characterized by an intake of high fat, usually adequate protein, and always strongly restricted carbohydrate amounts. Its use leads to alteration in the energy metabolism and the use of ketone bodies (KBs), instead of glucose, as a primary energy source.^1−4^ During normal glycolysis, when a typical diet is consumed, glucose is converted to pyruvate, which is then transformed into acetyl-CoA.^5^ During consumption of KD, when an amount of glucose is limited, the glycolysis process is significantly reduced.^2,6^ In this case, fatty acids undergo oxidation, which leads to the production of acetyl-CoA from them.^1,6^ This compound takes part in the process of ketogenesis in the liver as a result of which three main KBs, acetone, acetoacetate, and β-hydroxybutyrate,^1,3,6^ are produced. Excess acetyl-CoA produced from fats cannot be used in the Krebs cycle; therefore, its remaining amount is converted to acetoacetate.^1,6^ In turn, acetoacetate may be spontaneously degraded to acetone or enzymatically converted by β-hydroxybutyrate dehydrogenase to β-hydroxybutyrate.^1,6^ Elevated ketone concentrations found in urine or serum testify about the state of ketosis and may be used as a marker of early compliance following dietary initiation.^3,7,8^

Many researchers suggest some therapeutic benefits related to a state of ketosis and point out potential applications of KD for the treatment of a variety of metabolic, oncologic, neurodegenerative, and psychiatric disorders.^9−11^ Although KD has been tailored to meet the needs of patients suffering from epilepsy,^3,9^ the literature evidence indicates that it can be successfully used to treat obesity,^12^ Alzheimer’s disease,^13^ traumatic brain injury,^14^ depression,^15^ or Parkinson’s disease.^11^

KD has been shown to have positive effects in the case of drug-resistant epilepsy in both children^16,17^ and adults.^7,8,18^ A particular challenge is still an effective and safe treatment of pregnant women with epilepsy as most of antiseizure drugs are teratogenic.^19^ In this case, any alternative treatments for the disease should be considered, and one of them may be KD. First, however, it is necessary to find out how such a dietary therapy may affect a developing fetus. A placenta acts as a filter that allows nutrients to pass from the mother to the fetus's blood.^20^ Herrera and Gómez-Coronado^21^ pointed out that KBs circulating in the mother's plasma can cross the placenta and reach the same level in the fetus. Furthermore, they may be used for brain lipid synthesis of developing offspring.^21^ However, according to our best knowledge, there is still a lack of medical evidence answering the question of whether in the case of higher levels of KBs present in the mother organism, the risk of ketoacidosis in the fetus increases. A case study of two pregnant women suffering from epilepsy, described by van der Louw and Williams,^22^ showed no negative impact of prenatal exposure to KD on child health and its nervous system development. The authors of the cited article, however, pointed out that further monitoring of children is warranted to identify possible long-term side effects of the therapy.^22^

Conclusions from the animal studies regarding an influence of the high-fat diet used in gestation on pups are not consistent.^9^ Discrepancies can be noted, among others, taking into account the body weight of the offspring. Some investigations showed that a maternal high-fat diet before and during pregnancy causes a significant up-regulation of placental nutrient transport and fetal overgrowth both in mice and rats.^23,24^ Another study showed that embryos, mothers of which were exposed to the KD diet during pregnancy were volumetrically larger in the middle of gestation, in comparison to their counterparts from the control group and then the volumetric decreases at a later stage were noticed.^25^ It has also been reported that such a diet used during pregnancy and early postnatal life may lead to alterations in the neonatal brain structure, including the areas of the cortex, hippocampus, corpus callosum, fimbria, lateral ventricles, hypothalamus, and medulla.^26^ The authors suggested that such anomalies could be associated with functional and behavioral changes in later postnatal life.^26^ Our previous investigation showed a reduction of the body mass and delays in the neurological development of the offspring from females fed during gestation with KD.^27^ However, discontinuation of the high-fat diet and introducing the standard one in mothers at the beginning of lactation resulted in the recovery of weight and neurological function of pups to the normal levels already on the postnatal day 14th.^27^

Our previous studies showed that the spatial distribution and the accumulation of main biological macromolecules within the brain differ between rats fed prenatally with ketogenic and standard laboratory diets.^28^ In 14-day-old offspring of KD-fed mothers, an increase in the relative level of compounds containing carbonyl groups as well as a decrease in the relative content of lipids and their structural changes in some areas of the brain were observed.^28^ Moreover, the chemical mapping of absorption bands specific to lipids showed that the surface of the internal capsule is smaller for these animals.

This study aims to identify topographic and quantitative elemental anomalies appearing in offspring brains as a result of maternal ketogenic diet (KD) treatment during gestation. To achieve this, we compared the male offspring of the female rats fed during pregnancy either with a ketogenic or standard laboratory diet. The study included the progeny at 2, 6, 14, 30, and 60 days of age, enabling us to monitor the progression of potential elemental changes and determine whether they are temporary or persistent. The elemental mapping of brain slices was performed using synchrotron radiation-induced X-ray fluorescence (SR-XRF) microscopy being a nondestructive, highly sensitive, and relatively fast technique of multielemental analysis. This method has proven to be very useful in our previous research on the epilepsy pathogenesis and progression,^29−32^ the neuroprotective and antiseizure effect of KD,^33−35^ and the elemental markers of brain injury and glioma development.^36^ Element-sensitive hard X-ray imaging, necessary to realize the purposes of the present paper, was conducted at the FLUO beamline^37^ of the KIT Synchrotron light source (Karlsruhe, Germany) and the used experimental conditions allowed us to track the spatial distribution and accumulation of phosphorus (P), sulfur (S), potassium (K), calcium (Ca), iron (Fe), and zinc (Zn) within the scanned tissue regions.

## Results and Discussion

Considering the safety of the use of KD by pregnant women, it is crucial to determine the potential effects of such dietary treatment on the offspring, among others, on the nervous system. In our previous investigations we focused on checking how KD used during gestation influences the body mass, neurological functions, and general state of pups^27^ as well as the distribution, accumulation, and structure of biomolecules in their brains.^28^ The purpose of this study was to verify if prenatally used KD has an influence on the topographic and quantitative elemental changes occurring in the offspring's brain with age.

**Figure 1:**
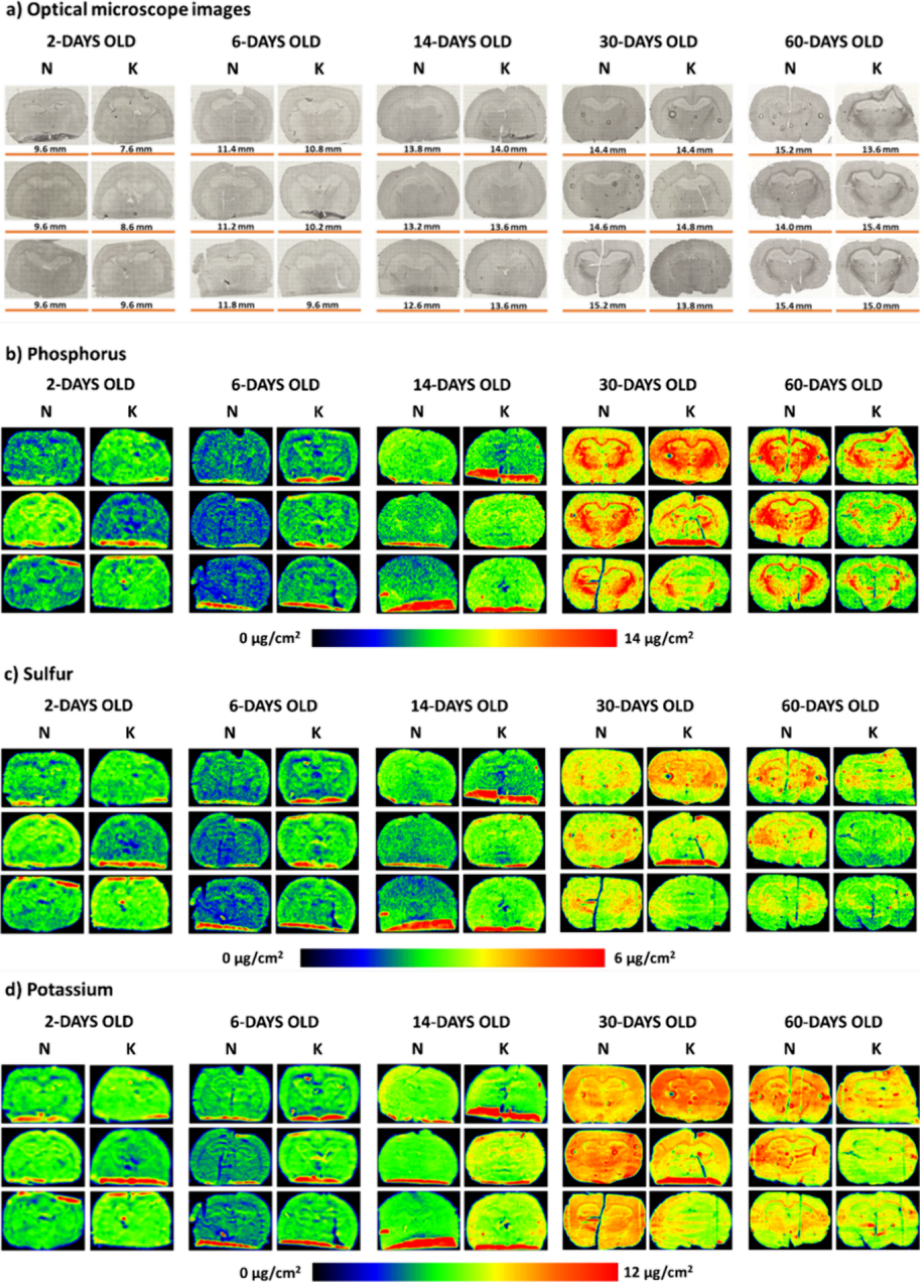
Microscope images (a) and distribution maps of P, S, and K (b–d, respectively) in the representative brain tissue slices taken from the rats of all the examined stages of postnatal development (2-, 6-, 14-, 30-, and 60-days old), the mothers of which were fed during pregnancy with the ketogenic (K) or standard fodder (N). Color scales below the maps express the elemental mass deposits in μ*g*/*cm*^2^.

**Figure 2:**
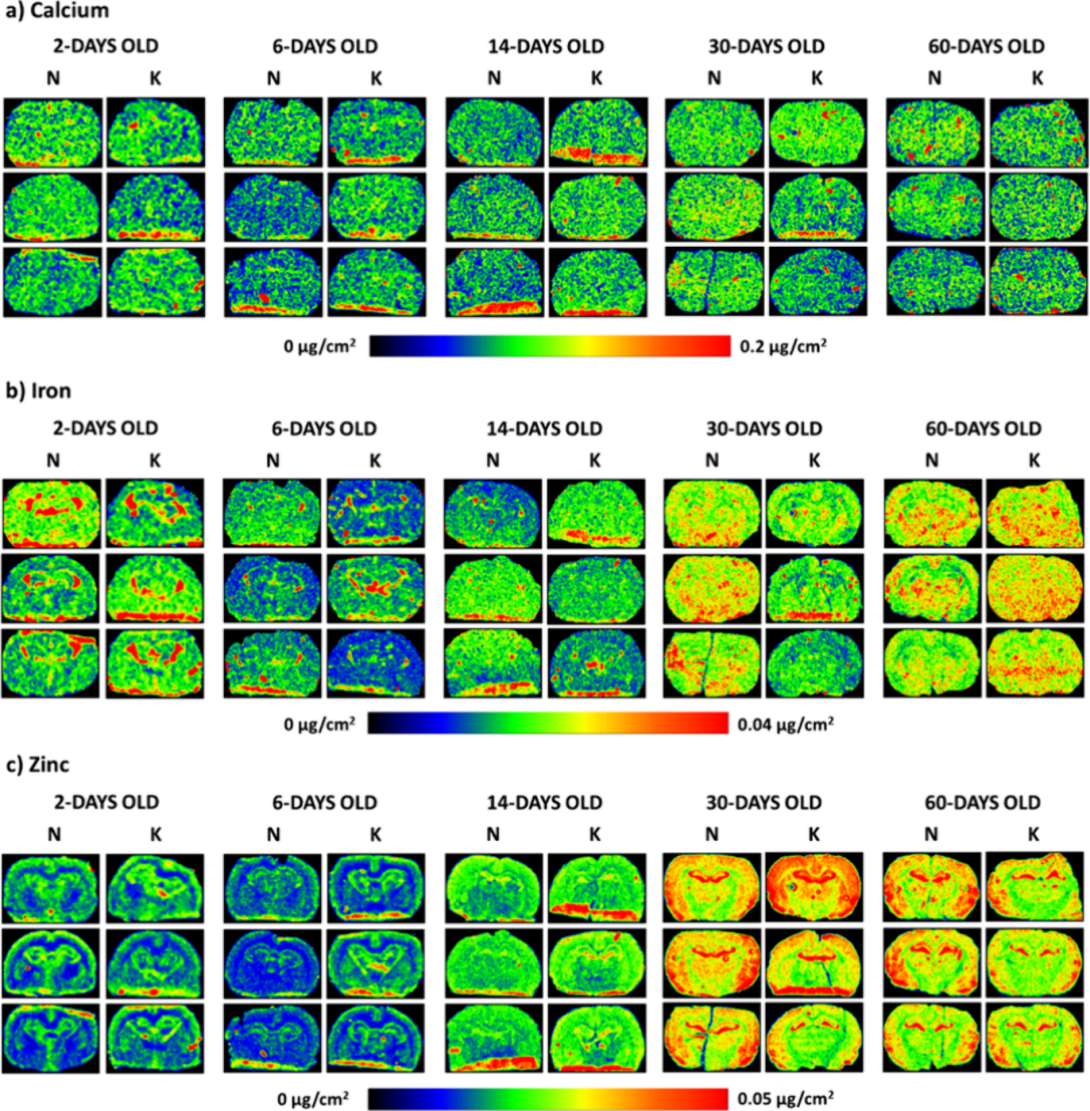
Distribution maps of Ca, Fe, and Zn (a–c, respectively) in the representative brain tissue slices taken from the rats of all the examined stages of postnatal development (2-, 6-, 14-, 30-, and 60-days old), the mothers of which were fed during pregnancy with the ketogenic (K) or standard fodder (N). Color scales below the maps express the element mass deposits in μg/cm^2^.

The results of the topographic elemental analysis from mapping the distributions of P, S, K, Ca, Fe, and Zn in the examined brain slices are shown in Figures 1 and 2. Additionally, in Figure 1, optical microscope images showing anatomical features of the examined tissues were presented.

The element distribution maps shown in Figures 1 and 2 indicate that the level of most of the examined elements is higher in the most mature brains (60-day-old animals) than in the initial stages of the postnatal development of the nervous system. The only exception here is Ca, the mass deposit of which, regardless of the mother’s diet during pregnancy, does not differ significantly among the rats at various ages. The most dynamic changes in the accumulation of the majority of elements in the brains were observed between the 14th and 30th days of the animals' life. In turn, their lowest levels were found in 6-day-old rats.

A qualitative comparison of the element distribution maps from the offspring of mothers fed prenatally with the ketogenic and standard diets indicates an influence of the dietary nutrition used during pregnancy on the level of low-Z elements in the brain. The rats, prenatally exposed to the high-fat diet, at the age of 6 and 14 days seemed to have a higher accumulation of P, S, and K than their corresponding control animals. The opposite tendency for the mentioned elements was observed for the adult (60-day-old) animals. In the 30- and 60-day-old offspring of the mothers fed with KD during pregnancy, a reduction of the surface of brain areas characterized by an increased P and S content, which histologically/morphologically correspond to white matter structures, was noticed. The Fe distribution maps indicated a lower content of this element in the brains of 30-day-old animals whose mothers received high-fat fodder. In turn, Zn distribution maps revealed that the use of KD during pregnancy leads to a temporary increase in the level of this element in the hippocampal formation of the young offspring (6- and 14-day-old).

**Figure 3:**
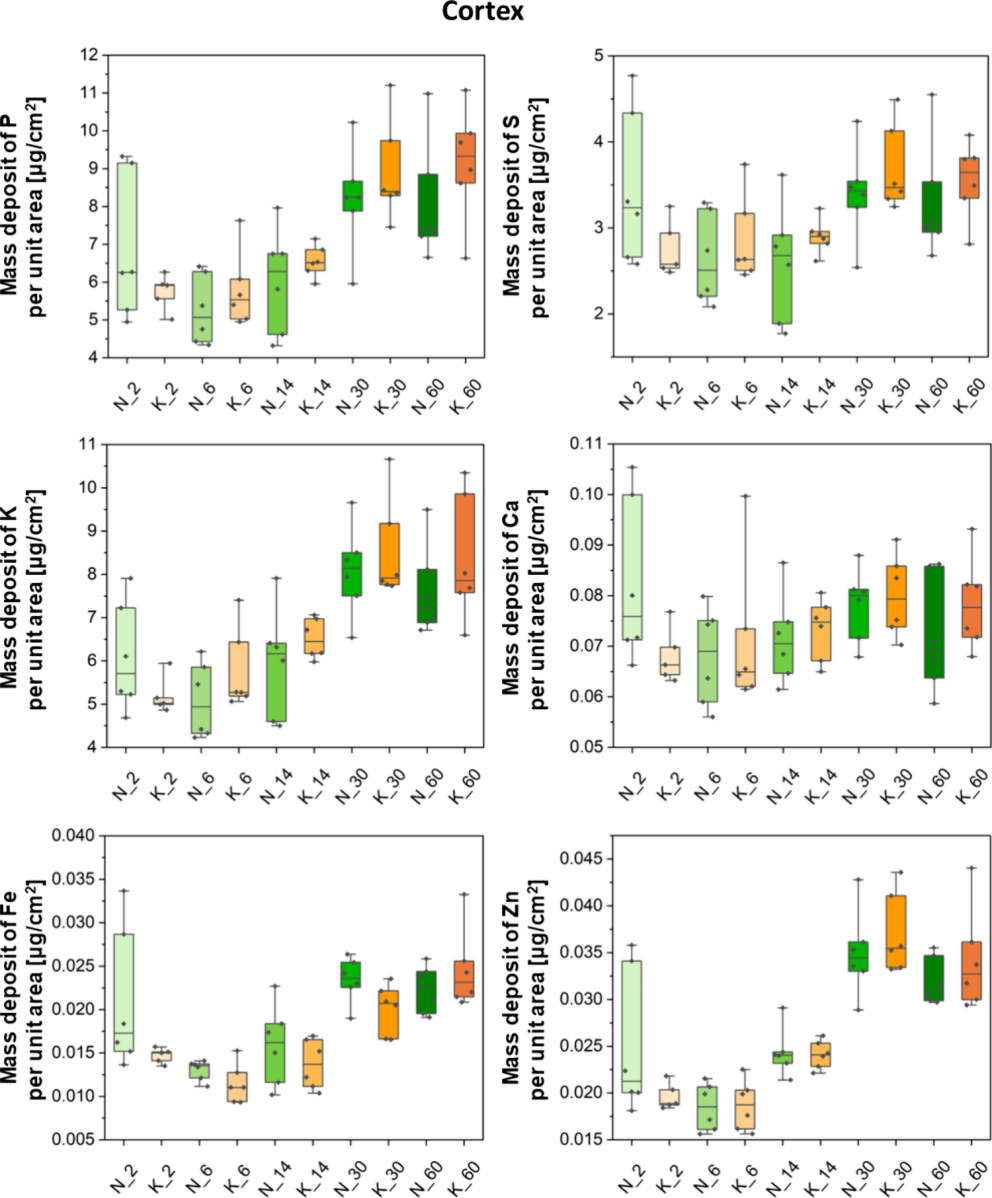
Box-and-whisker plots presenting the median, minimal, and maximal values as well as interquartile spans of the mass deposits of P, S, K, Ca, Fe, and Zn in the cortex determined for the experimental (K) and control (N) groups. The 2-, 6-, 14-, 30-, and 60-days old rats were taken into account in the analysis. No statistically significant differences (Mann–Whitney *U* test, 95% confidence level) were found between the K and N groups of the rats at a given age.

**Figure 4:**
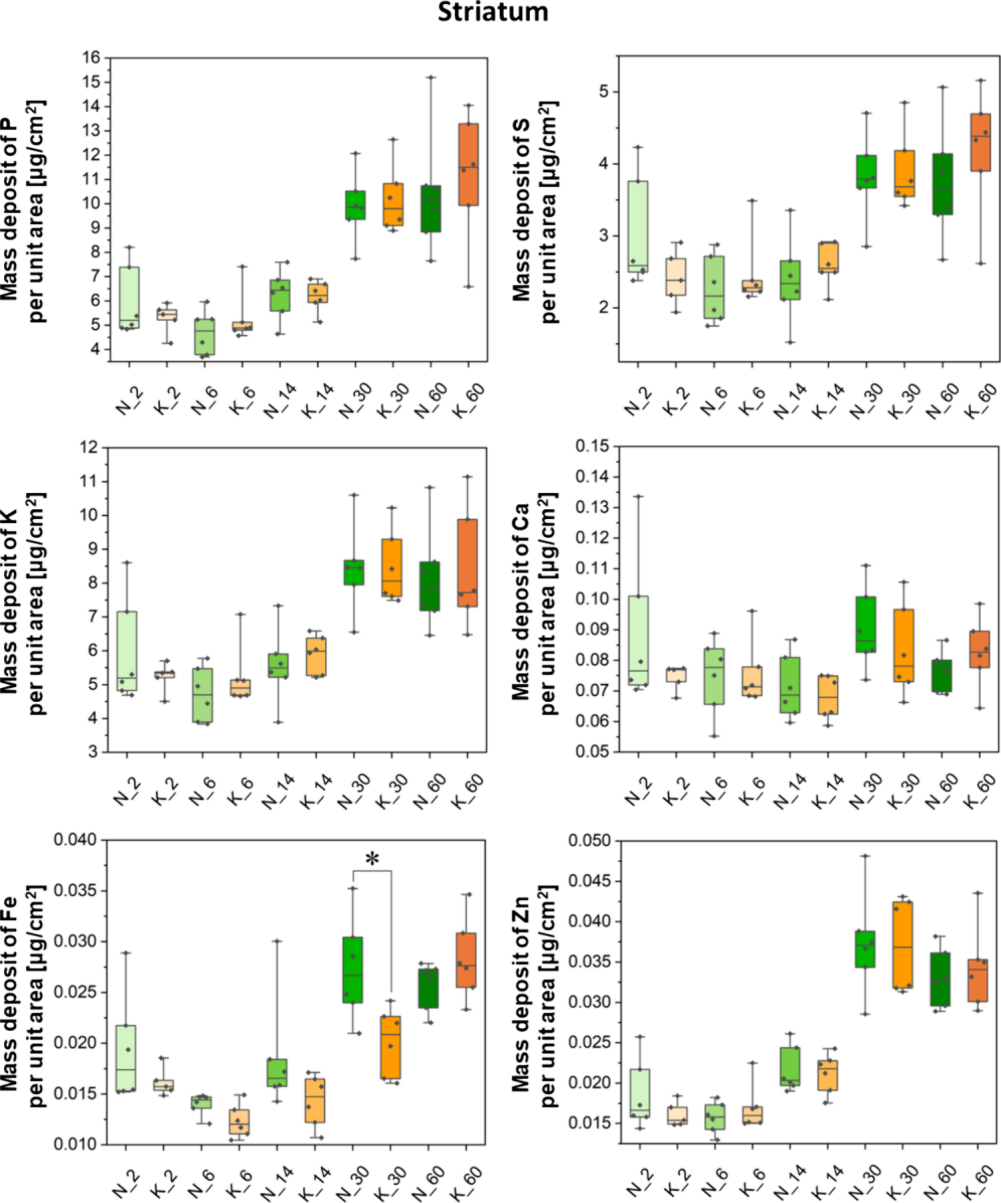
Box-and-whisker plots presenting the median, minimal, and maximal values as well as interquartile spans of the mass deposits of P, S, K, Ca, Fe, and Zn in the striatum determined for the experimental (K) and control (N) groups. The 2-, 6-, 14-, 30-, and 60-day-old rats were taken into account in the analysis. Statistically significant difference(s) (Mann–Whitney *U* test, 95% confidence level) between the K and N groups at a given age was(were) marked with *.

**Figure 5:**
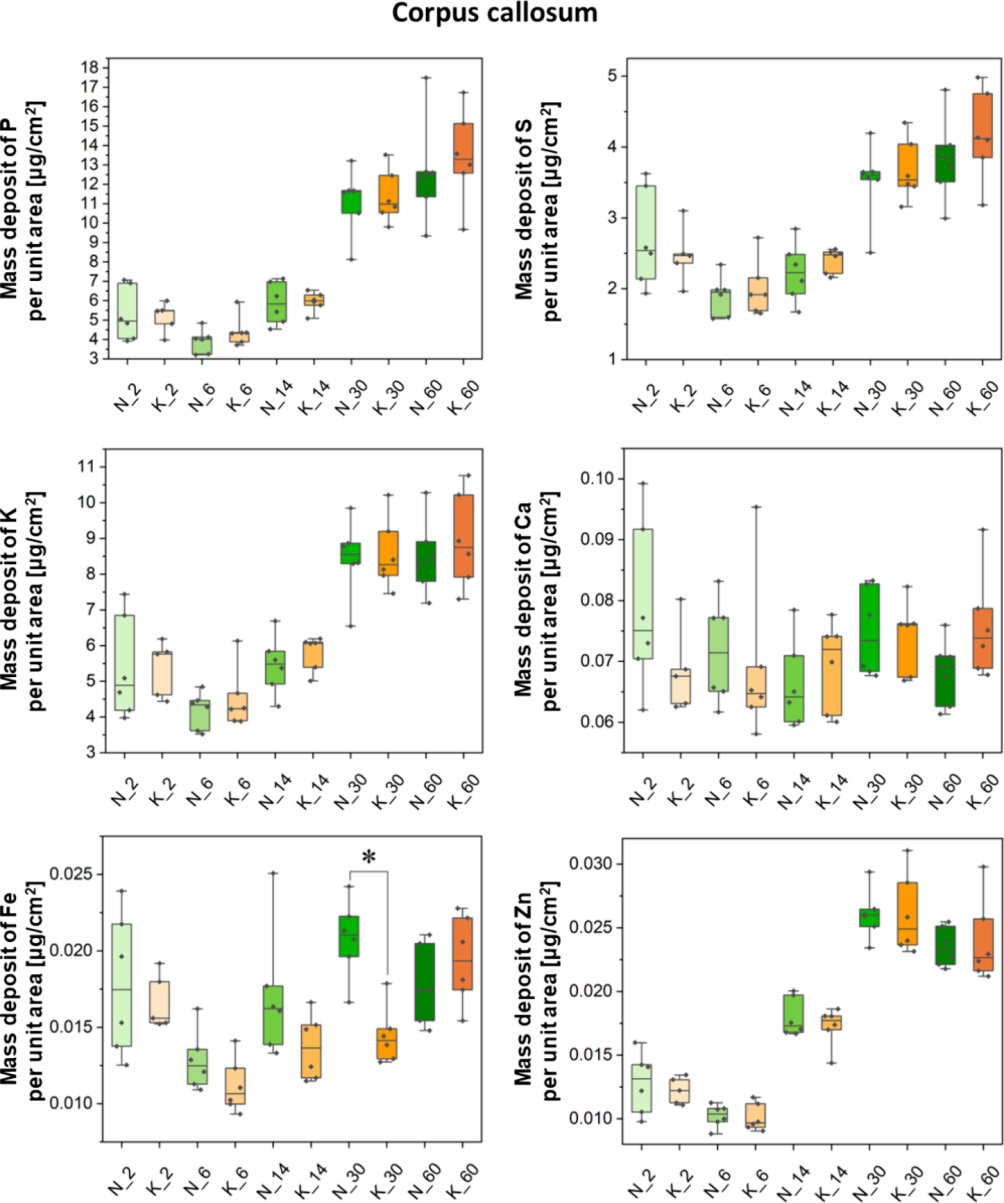
Box-and-whisker plots presenting the median, minimal, and maximal values as well as interquartile spans of the mass deposits of P, S, K, Ca, Fe, and Zn in the corpus callosum determined for the experimental (K) and control (N) groups. The 2-, 6-, 14-, 30-, and 60-day-old rats were taken into account in the analysis. Statistically significant difference(s) (Mann–Whitney *U* test, 95% confidence level) between the K and N groups at a given age was(were) marked with *.

**Figure 6:**
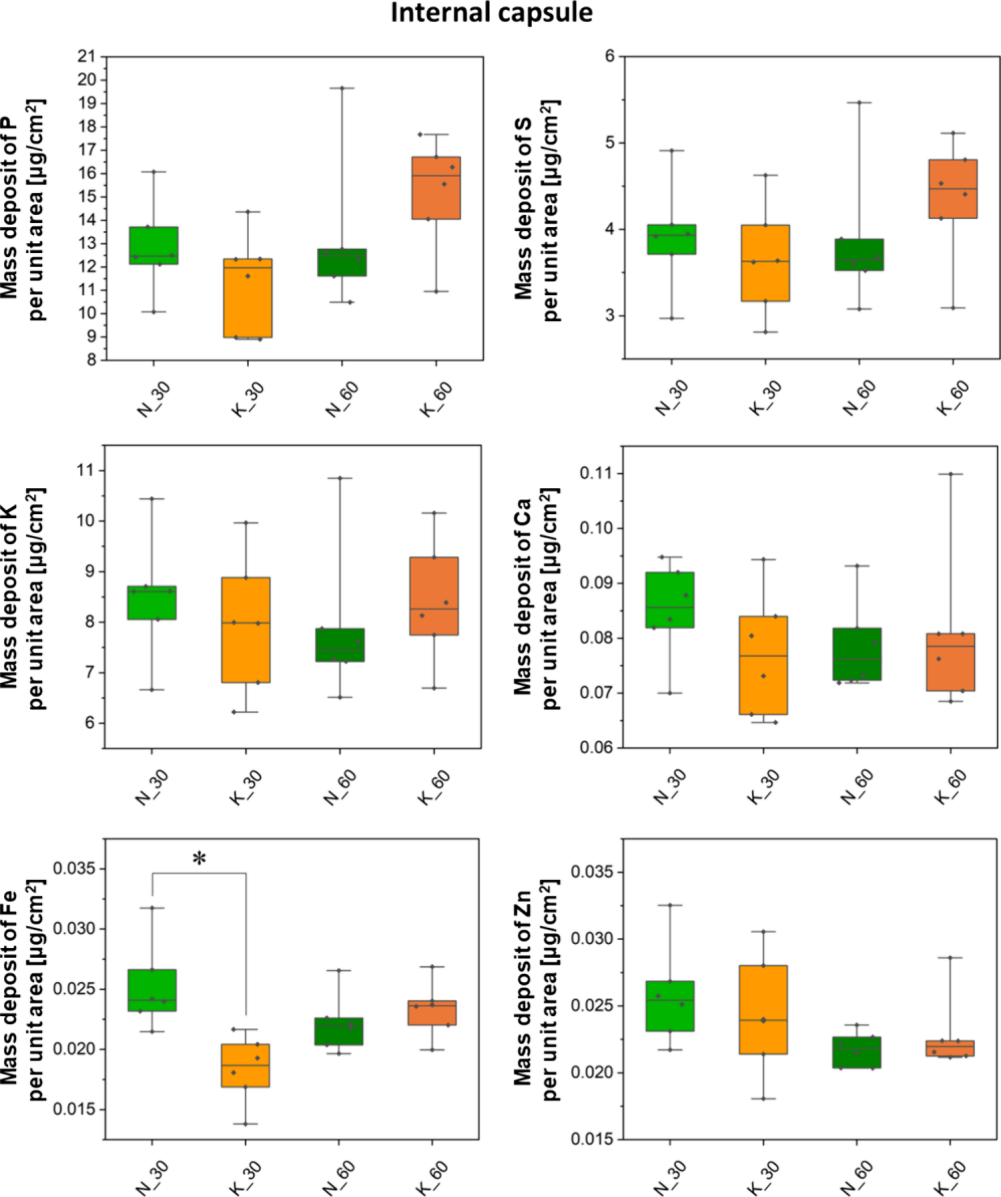
Box-and-whisker plots presenting the median, minimal, and maximal values as well as interquartile spans of the mass deposits of P, S, K, Ca, Fe, and Zn in the internal capsule determined for the experimental (K) and control (N) groups. The 30- and 60-day-old rats were taken into account in the analysis. Statistically significant difference(s) (Mann–Whitney *U* test, 95% confidence level) between the K and N groups at a given age was(were) marked with *.

Besides the topographic analysis giving insight into the global element changes occurring in the brain as a result of postnatal development and prenatally used KD, a more detailed quantitative analysis was performed for the selected brain areas. These were the cortex, striatum, and corpus callosum and, in the case of the older animals, also the internal capsule. Results of quantitative comparisons between the experimental KD-fed groups (K_2, K_6, K_14, K_30, and K_60 where the numbers denote the age) and their corresponding control animals (N_2, N_6, N_14, N_30, and N_60) are shown in a form of box-and-whisker plots in Figures 3–6. Furthermore, to follow the dynamics of elemental changes occurring during postnatal brain development, the statistical significance of the differences in the elemental accumulation between the subsequent time points was verified, and the obtained results are presented in the Supporting Information in Figure S1.

The Mann–Whitney *U* test was performed in order to identify statistically relevant differences in the accumulation of the examined elements in the selected brain areas. Its results showed only some temporary differences in the Fe mass deposits between the experimental and control animals. In 30-day-old rats that were exposed prenatally to the KD, a lower level of Fe in the striatum and in both studied white matter structures (namely, corpus callosum and internal capsule) was noticed. The aforementioned effect, however, was not observed in adult animals.

**Figure 7:**
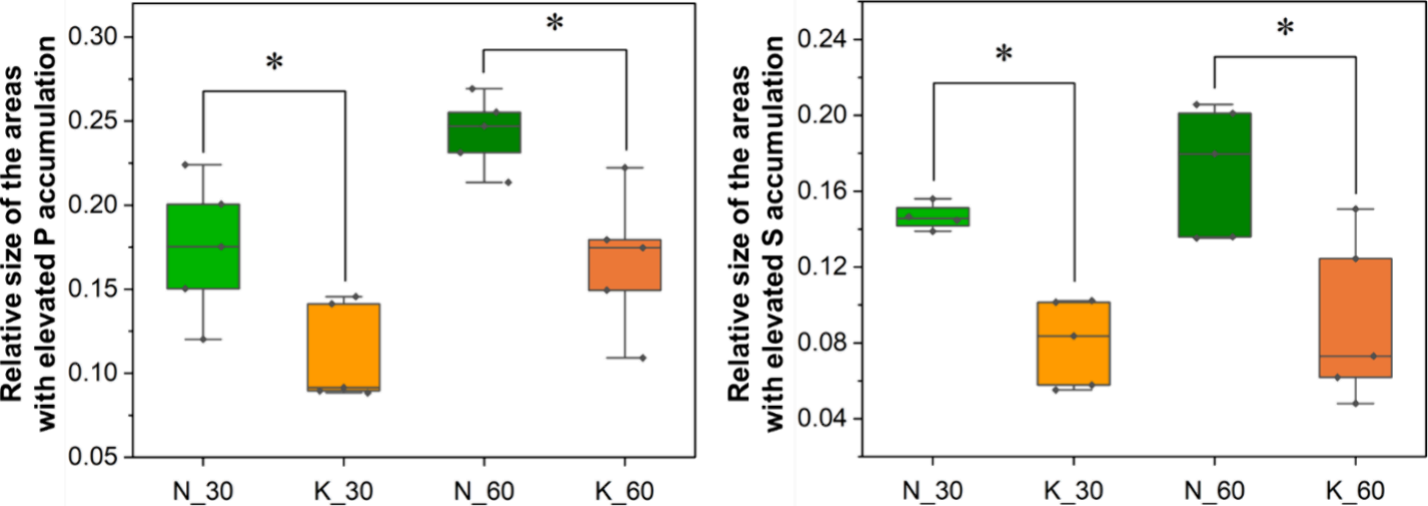
Box-and-whisker plots presenting the median, minimal, and maximal values of the relative sizes of the areas characterized by the increased P and S accumulation for the 30- and 60-day-old experimental and control rats (K and N groups, respectively). Statistically significant difference(s) (Mann–Whitney *U* test, 95% confidence level) between the K and N groups at a given age were marked with *.

The previously performed topographic analysis suggested also the existence of the differences in the size of the areas characterized by the increased P and S levels and corresponding to the structures of white matter between the older offspring of mothers fed during pregnancy with the ketogenic and normal diet. Figure 7 presents box-and-whisker plots showing the median, minimal, and maximal values of the relative sizes of the areas characterized by the increased P and S accumulation for the 30- and 60-day-old experimental and control rats (K and N groups, respectively). The method of determining these relative sizes is presented in the Materials and Methods chapter. As one can easily notice in Figure 7, the statistical analysis performed confirmed the prior qualitatively observed differences.

Statistical analysis confirmed that the content of P in rat brains increases mainly between the 14th and 30th days of their postnatal life and it does not change at a later stage of the development. The consumption of KD by mothers during pregnancy seems to affect the level of this element in the brains of the offspring. Qualitative analysis showed that the rats at the age of 6 and 14 days, exposed prenatally to the high-fat diet, showed in general a higher accumulation of P than the corresponding control animals, in turn, the opposite relationship was observed for adult ones. However, the statistical analysis performed for selected brain regions did not reveal any statistically significant differences. Additionally, the subarea highly abundant with P, corresponding morphologically to the white matter, was significantly smaller in the case of the 30- and 60-day-old pups of the KD-fed mothers. A similar relationship was also observed in our previous paper^28^ with respect to the spatial distribution of lipids in the 14-day-old offspring of high-fat fodder fed rats. The subarea of the brain characterized by an increased intensity of lipid bands (corresponding to the internal capsule of the white matter) was smaller in animals when the mothers during pregnancy were treated with KD.

The white matter constitutes well-vascularized clusters of nerve fibers. These fibers are mostly axons surrounded by myelin sheaths which, in the brain, are formed by oligodendrocytes.^38−40^ Myelin contains 70–85% of lipids and the most abundant of them are galactocerebroside, sphingomyelin (containing predominantly a phosphocholine as a headgroup), and cholesterol.^41,42^ The observed in the pups of KD-fed mothers correlated abnormalities in the distributions of P and lipids may mirror the changes in the accumulation of sphingomyelin and, the same, point at the disturbances in the structure and integrity of the myelin. Recently, more and more attention has been focused on the role of myelin in the central nervous system and its most known functions are the acceleration of nerve impulse conduction and increasing energy efficiency in this process.^40,42^ Furthermore, myelinating glial cells, such as oligodendrocytes, present in the myelin sheath provide physical and chemical protection to axons, which make them more resistant to damage, but also regulate their ionic environment and fuel their energy demands with metabolites.^43^ There is also growing evidence that the myelin sheath provides a trophic support to the axons.^41,44^ Facilitating efficient communication between nerve cells, the myelin is crucial for the proper action of the nervous system and it plays a crucial role in cognition, learning, and even human behavior.^40,45,46^ However, to check how the observed changes in the white matter structure affect the function of the nervous system or animal behavior, further long-term observation of the offspring is necessary.

The content of S and K remained at a relatively constant level in the initial stages of brain development, and a noticeable change in the accumulation of these elements occurred on the 30th day of animal life. The qualitative topographic analysis showed, moreover, that similarly as in the case of P, the level of S and K presented the opposite pattern of changes induced by prenatal exposure to KD in the case of younger and older animals. One of the reasons for the higher content of low-Z elements in the brains of the younger offspring from the females fed with the high-fat diet could be a higher content of these elements in the KD than in the standard laboratory diet. However, the analysis of the element composition of the fodders performed by us indicates exactly the opposite relationship for P and S, in which the concentration was lower in the high-fat than in the standard diet.^47^ The content of K in both diets was at a similar level. However, because of the lower fodder mass consumed by the rats on KD, the intake of K through these animals was smaller compared to that of controls. It also should be noted that in the mothers fed with KD during pregnancy, the standard diet was introduced at 2 days of postpartum and continued during the whole time of lactation, so the potential KD influence would be associated with the period before postpartum.

S is an essential component that influences the function of several bioactive molecules. Sulfatide, a sulfated form of galactocerebroside, is an important sulfoglycolipid found on the extracellular leaflet of myelin.^48,49^ Most of the sulfatide in the nervous system is present in myelinating cells, namely oligodendrocytes in the central nerves and Schwann cells in the peripheral ones.^50^ The literature evidence points out that these sulfoglycolipids may play an important role in the differentiation of myelinating cells, formation of the paranodal junctions, and general myelin maintenance.^49,51^ They are involved in the formation of membrane microdomains (lipid rafts), where together with cholesterol and raft-associated proteins play roles in different myelin functions.^49^ Moreover, sulfatides participate in a variety of cellular processes such as protein trafficking, axon-myelin interactions, neural plasticity, and immune response.^52^ The research indicates that although the lack of sulfatide does not interfere with the processes related to the myelin formation or cause drastic changes in its structure, it does prevent the maintenance of the normal myelin sheath in adult mice.^53,54^ The colocalization, we found in the present paper, between the areas of the increased S content and the structures of the white matter may be a result of the presence of sulfatides from the myelin sheaths. Taking into consideration the above-mentioned dependences, the reduction of this surface in the offspring of the KD-fed mothers may testify about white matter abnormalities.

We noticed a reduction in the level of Fe in the 30-day-old rats, mothers of which were fed with KD during pregnancy. Although excessive Fe accumulation in the brain appears to be much more dangerous than element deficiency, any imbalance in micronutrients should be monitored. On the other hand, the nature of the anomalies, we found in the present paper, seems to be transient as no similar relationship was observed in the older, 60-days-old animals. The research based on animal models showed that adequate Fe levels support the proper neurological and cognitive development while its fetal and neonatal deficiency may reduce the oxidative metabolism in the hippocampus and frontal cortex, increase concentrations of intracellular neuronal glutamate, reduce dopamine distribution in the striatum, and alter the fatty acids and myelin profiles in brains.^55−58^ Fe concentration in the brain increases with age, because of the constant intake and the slow exchange of this element during the whole life.^59^ Our results collected in the present investigation showed that the level of Fe, for both the experimental and control groups, was the lowest in the 6-day-old animals, and it slowly increased in subsequent time periods.

Fe is a micronutrient that is needed for the proper functioning of the nervous system. It has the ability to cross the blood-brain barrier (BBB) and participate in the generation of neurotransmitters and axonal myelination.^59^ Furthermore, various metabolic processes, including the synthesis of DNA, which is crucial for cell division and growth, demand this element.^36,60^ Despite the many positive roles of Fe in the nervous system, the element may also be potentially toxic for living cells by catalyzing Fenton’s reaction, which leads to the creation of highly reactive hydroxyl radicals, intensifying the effect of the oxidative stress.^31,59^ The decreased level of Fe, which we found in the 30-day-old offspring of the high-fat fodder-fed mothers, may be related to the above-mentioned changes, namely, the reduction of the relative surface of the other elements in the white matter in the older rats exposed prenatally to KD. However, at this stage of our investigation, it is not possible to answer the question of whether or not the brain structural changes are induced by the diminished level of the element or if the diminished deposits of Fe are a result of intensified processes of myelination and formation of neural connections.

Among all of the investigated elements, the most dynamic changes during the animal postnatal development were found for Zn. The lowest mass deposit of this element was observed in the brains of the 6-day-old rats. The level of Zn increased during the next two observation periods and then remained at a similar level between the 30th and 60th days of the animal life. The mass deposit of Zn within the hippocampal formation of the control rats diminished between the second and sixth days of postnatal development. Afterward, its level increased to reach the highest quantity in the 60-day-old animals, which is in agreement with our previous study.^61^ The comparison between the offspring of the mothers fed during pregnancy with the high-fat and standard fodder showed a higher Zn accumulation in the hippocampus of the 6- and 14-day-old rats prenatally exposed to the KD. The increase of Zn accumulation in particular areas of the brain at the beginning of its development may testify to the reorganization of GABA-ergic innervation, sprouting of mossy fibers, and/or functional changes of nervous tissue.^61^ The higher level of the element observed temporarily within the hippocampal formation of the animals exposed prenatally to KD may point to the intensification of the processes of nervous tissue reorganization within this brain area.

Summarizing, elements such as P, S, K, Ca, Fe, and Zn perform many important functions in the brain, so it is important to monitor potential changes in their levels and distributions in the offspring nervous system that may be caused by the use of KD during pregnancy. In this paper, we showed that X-ray fluorescence microscopy can be successfully employed to track the element changes occurring in the brain during its postnatal development as well as those induced by prenatal exposure to high-fat fodder. In view of our present results, KD implemented in mothers influences the element content in the brains of their offspring. Moreover, our investigation showed some topographical abnormalities in the brains of the oldest rats exposed prenatally to KD. For these animals, a reduction of brain area characterized by an increased P and S level and histologically/morphologically corresponding to the structures of white matter were noticed. In order to confirm or exclude different influences of the prenatally used KD on the offspring depending on their gender, it is worth extending the investigation in the future to female pups. Further research on mechanisms of the observed phenomena and their possible health consequences is still necessary in order to increase the chances for the implementation of such dietary treatment of epilepsy in pregnant women.

## Materials and Methods

### Animals

The rats investigated in this study originated from the Laboratory of Experimental Neuropathology at the Institute of Zoology and Biomedical Research (Jagiellonian University, Krakow, Poland). A culture of animals as well as all animal-use experiments was also conducted there. This was done in accordance with permission 122/2015 of the First Local Ethical Committee and with international standards. In the study, the male rats of the Wistar strain at different ages, which had been born by females receiving during their gestation the ketogenic or standard diet (experimental and control groups, respectively), were used. Both diets were maintained during the entire period of pregnancy. In the case of the mothers of the control group, the standard laboratory diet was continued during lactation. In turn, in the case of the previously KD-fed females, a normal diet was introduced 2 days after labor. The offspring were nourished with maternal milk until 21 days of postnatal life, after which they were transferred to individual cages and provided with a standard diet. The details of the experiment were described in our previous papers.^27,28^ The present study included 5 stages of the offspring development, namely, 2-, 6-, 14-, 30-, and 60-day-old rats, and the typical number of animals in each group was 6.

### Ketogenic and Standard Laboratory Diet

KD with long-chain fatty acids (ssniff, EF R/M with 80% Fat) and the standard laboratory diet in the form of Labofeed (Morawski, Inc.) were used in the study. The content of main nutrients (defined by the producers of the fodders) as well as the major, minor, and trace elements (determined by us using the TXRF method) in both diets was presented in our previous works.^28,47^

### Sample Preparation

On the 2nd, 6th, 14th, 30th, and 60th days of postnatal life (depending on the group), the animals were anesthetized with Morbital (Biowet) and perfused with a physiological saline solution of high analytical purity. After that, the brains were extracted from the skulls, deeply frozen in liquid nitrogen, and cut with a cryomicrotome into slices with a thickness of 20 μm. From each brain, the slice containing the dorsal part of the hippocampal formation was taken (Figure 10 A), placed on a stretched Ultralene foil, and freeze-dried.

### SR-XRF Element Imaging

To visualize the mass deposits of the elements of our interest within the brain slices and to perform subsequently a topographic and quantitative elemental analysis, SR-XRF microscopy was applied. The brain tissues were scanned at the FLUO beamline installed at the KIT synchrotron light source in Karlsruhe, Germany. The energy of the exciting X-ray beam was 17 keV while its size was 200 μm (v) × 200 μm (h). Such a relatively large X-ray beam was employed for raster scanning of the whole-area tissue slices with a step size of 200 μm in both directions and an acquisition time for a single XRF spectrum of 10 s.

### Qualitative, Topographic, and Quantitative Element Analysis

**Figure 8:**
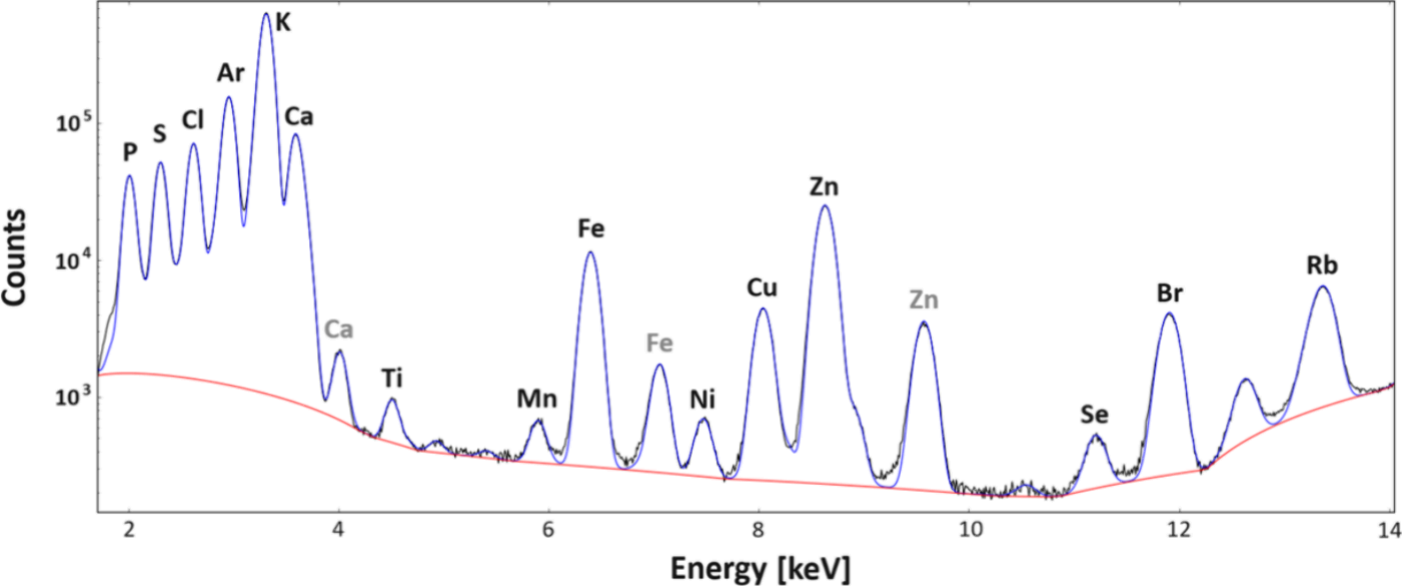
Typical cumulative X-ray fluorescence spectrum (black line) obtained for a brain tissue sample taken from a 60-day-old control rat. The fitted curve and its background are marked with blue and red line, respectively. The analytical Kα lines of the elements were marked in black, while Kβ lines were signed in gray.

The content of P, S, K, Ca, Fe, and Zn was determined in brain samples. Although, as one can see in Figure 8, other element X-ray lines were also detected in the cumulative XRF spectrum of tissue. Cl was excluded from further analysis because a NaCl solution was used for perfusion. Ar came from the ambient air, as the element imaging was not done in vacuum. In turn, Ti, Mn, and Ni in the cumulative XRF spectrum were of a nontissue but instrumentation origin. The remaining elements (Cu, Se, Br, and Rb), not taken into account in topographic and quantitative analysis, were below the limits of their detection for some of the examined pixels or tissue samples and therefore were not the subject of further analysis.

The two-dimensional topographic analysis of the elements of interest was based on mapping of their mass deposits in brain slices. The mass deposit per unit area of the element (*M*_*T*_) was calculated for each pixel of the map in accordance with eq 1):
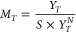
1

*M*_*T*_ is the mass deposit per unit area of the examined element in the tissue sample [μg/cm^2^].

*Y*_*T*_ is the net peak area of the *K*α line of the measured element for the tissue sample [cts].

*S* is the sensitivity for the measured element [cm^2^/μg].

*Y*_*T*_^*N*^ is the incoming X-ray beam normalization for the tissue sample [cts].

The sensitivities *S* for the measured elements were calculated on the basis of measurements of the Micromatter Technologies Inc. (Surrey, Canada) XRF calibration standards (RbI, Se, Cu, Ti, Fe, CaF_2_, GaP, SrF_2_, CsBr, ZnTe, and KCl) and using formula 2:
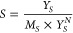
2

*Y*_*S*_ is the net peak area of the *K*α line of the measured element for the standard sample [cts].

*M*_*S*_ is the mass deposit per unit area of the analyzed element in the standard sample [μg/cm^2^].

*Y*_*S*_^*N*^ is the incoming X-ray beam normalization for the standard sample [cts].

**Figure 9:**
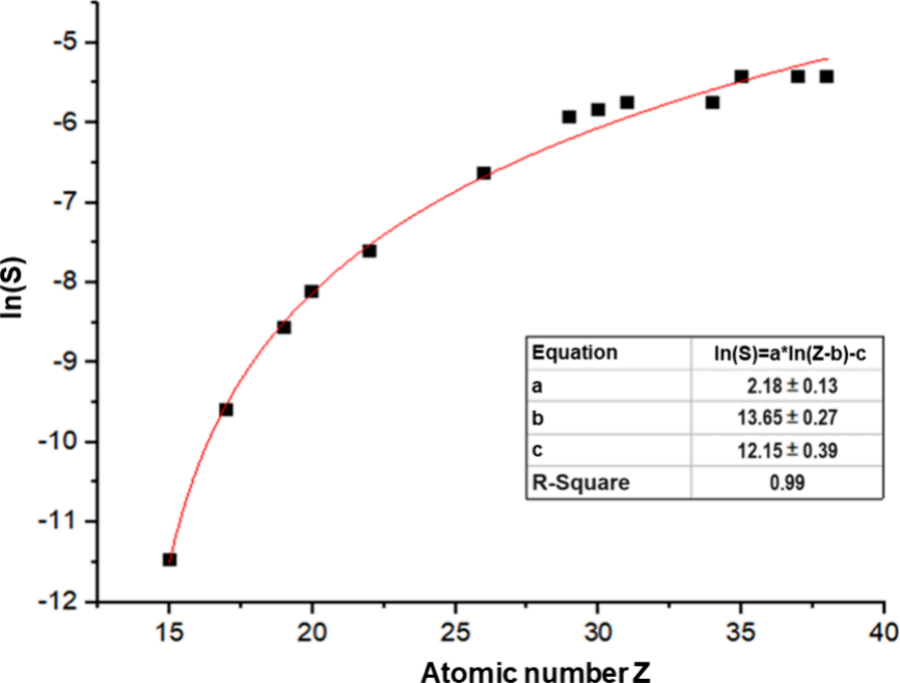
Sensitivity curve calculated on the basis of the micromatter XRF calibration standards.

For the XRF spectra normalization an electric charge accumulated within a semiconductor X-ray transparent diode placed upstream of the tissue/standard samples was used. The net peak areas of the *K*α lines for the selected elements were determined with the PyMca software^62^ (version 5.1.2). Once the sensitivities *S* were calculated, a calibration curve (Figure 9) was fitted according to eq 3 with the use of OriginPro (OriginLab Corporation, Northampton, MA, USA) software.
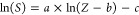
3*Z* is the atomic number of the measured element, *a*, *b*, and *c* are the parameters of the calibration curve.

**Figure 10:**
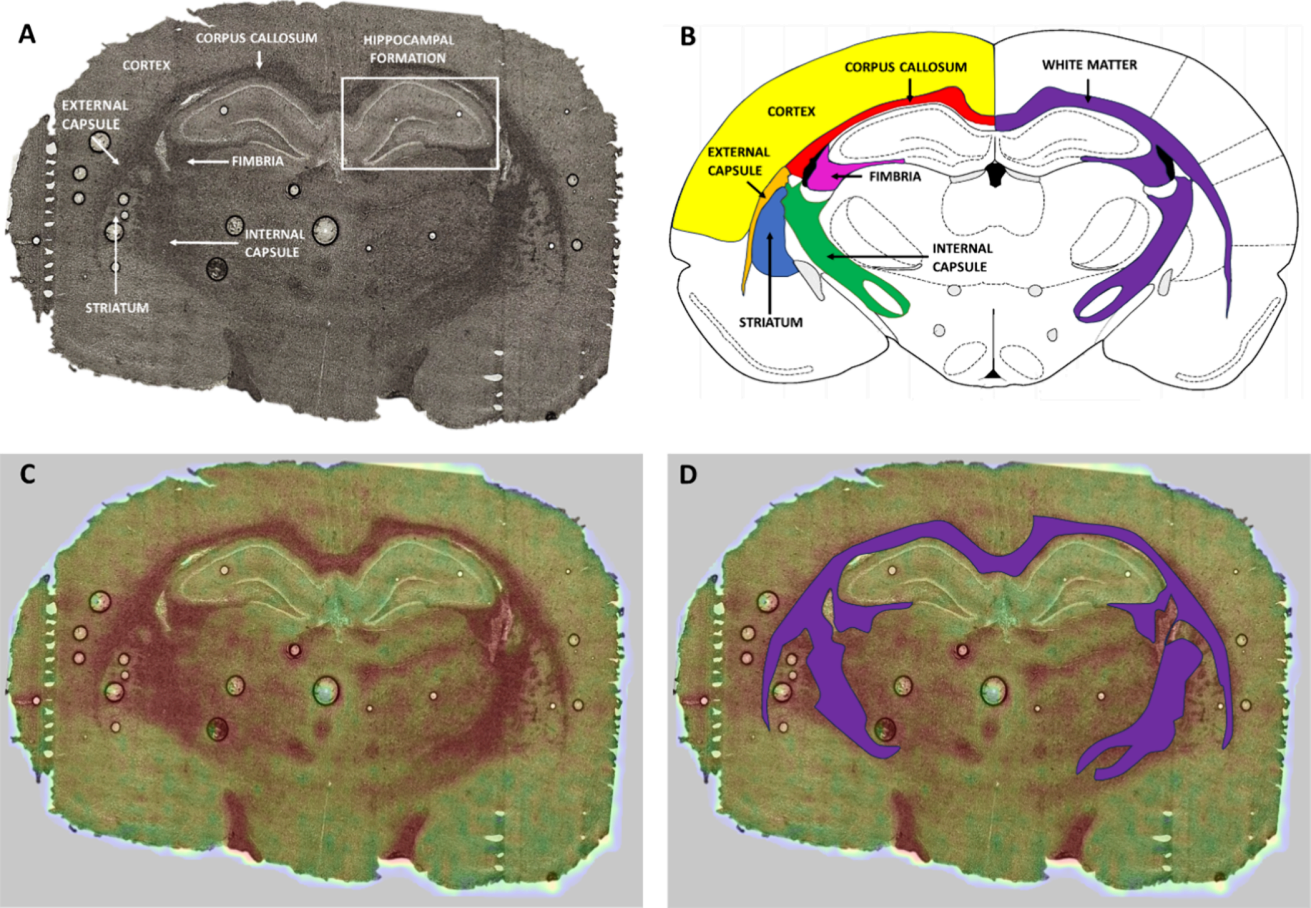
Comparison of the localization of selected regions (cortex, striatum, hippocampal formation and structures of white matter: corpus callosum, fimbria, internal capsule, and external capsule) in the microscopic image of an exemplary brain slice taken from 60-days old rat (A) with the graphics, based on the Paxinos and Watson anatomical atlas,^65^ presenting these brain areas in the corresponding coronal diagram (B). Blend of the microscopic image of the brain with the map of P distribution (C) and definition of the region taken into account in calculations of the relative size of the area characterized by the increased P content (D).

Once the element mass deposits per unit area for all the pixels of a particular map were calculated, the distributions of P, S, K, Ca, Fe, and Zn were drawn for the examined brain samples. The obtained maps were, then, compared with the corresponding microscopic pictures of the tissues in order to identify in them the important brain regions: cortex, striatum, hippocampal formation, and structures of white matter: corpus callosum, fimbria, internal capsule, and external capsule. For subsequent quantitative analysis, the cortex, striatum, and corpus callosum were selected. These regions were identified even for younger animals, and their size allowed for the collection of the number of data points allowing reliable quantitative analysis. In the case of older rats (30- and 60-day-old), the internal capsule was also included for further analysis as it was well visible in tissues taken for these age groups. In Figure 10 A,B, the localization of the mentioned regions within an exemplary brain slice was compared with the graphics based on the Paxinos and Watson rat brain anatomical atlas.

In order to perform the quantitative element analysis, for each examined slice, the average mass deposits of P, S, K, Ca, Fe, and Zn in the cortex, striatum, and selected structure(s) of the white matter were calculated. The average mass deposits were based on 50 randomly chosen points from particular areas (excluding some artifacts e.g. local contaminations of tissue, air bubbles formed between the tissue and the Utralene foil substrate, and tissue-free places within the scanned areas) and then employed for further statistical analysis. A nonparametric Mann–Whitney *U* test was applied to verify the significance of the differences between the animals exposed prenatally to KD and controls at the appropriate stage of postnatal development. The nonparametric *U* test is the right tool for statistical analysis because our data could not meet the assumptions about normality, homoscedasticity, and linearity, which are necessary for the use of parametric test.^63,64^ For the statistical analysis, OriginPro software was used, and the significance level was 5%.

The performed topographic analysis suggested the existence of the differences in the size of the areas characterized by the increased P and S levels between the older offspring of mothers fed during pregnancy with a ketogenic and normal diet. Histologically/morphologically these areas correspond to the structures of white matter. To verify this observation, the relative (compared to the whole brain slice) sizes of brain areas showing elevated P and S accumulation and corresponding white matter concentrations were determined. This was done independently for all the animals at the age of 30 and 60 days. Figure 10 C illustrates the methodology used to identify regions with elevated phosphorus and sulfur levels corresponding to white matter, which were included in the quantitative analysis. The sizes of the areas characterized by increased accumulation of mentioned elements as well as the surfaces of entire brain slices were determined with ImageJ software (version 1.52a, NIH, USA). Then, with the Mann–Whitney *U* test (95% confidence level), the ratios of these surfaces for the experimental and control rats were compared.

## Supporting Information Available

(Figure S1) Dynamics of elemental changes occurring in selected brain areas during postnatal development in the offspring of mothers fed during pregnancy with the ketogenic or standard fodder (PDF)
