# Supplementary material for: Element Changes Occurring in Brain Point at the White Matter Abnormalities in Rats Exposed to the Ketogenic Diet During Prenatal Life
Source: ACS Chem Neurosci. 2024 Oct 23;15(21):3932–44. doi: 10.1021/acschemneuro.4c00283 (PMC11587514; doi:10.1021/acschemneuro.4c00283)
Supplement: Supplementary file 1 — cn4c00283_si_001.pdf [file cn4c00283_si_001.pdf]

Supplementary Materials

Element changes occurring in brain point at the white matter abnormalities in rats exposed to the ketogenic diet during prenatal life

Marzena Rugieł[1#], Zuzanna Setkowicz[2#], Mateusz Czyzycki[3], Rolf Simon[3], Tilo Baumbach[3,4], Joanna Chwiej[1*]

[1] Faculty of Physics and Applied Computer Science, AGH University of Krakow, Al. Mickiewicza 30, 30-059 Krakow, Poland
[2] Institute of Zoology and Biomedical Research, Jagiellonian University, Gronostajowa 9, 30-387 Krakow, Poland
[3] Institute for Photon Science and Synchrotron Radiation, Karlsruhe Institute of Technology, Hermann-von-Helmholtz-Platz 1, D-76344 Eggenstein-Leopoldshafen, Germany
[4] Laboratory for Applications of Synchrotron Radiation, Karlsruhe Institute of Technology, Kaiserstr. 12, D-76131 Karlsruhe, Germany

N K

Phosphorus

Cortex	→	→	↗	→	Cortex	→	→	↗	→
Striatum	→	↗	↗	→	Striatum	→	→	↗	→
Corpus callosum	→	↗	↗	→	Corpus callosum	→	↗	↗	→
Internal capsule	—	—	—	→	Internal capsule	—	—	—	↗

2 6 14 30 60 2 6 14 30 60

Sulfur

Cortex	→	→	→	→	Cortex	→	→	↗	→
Striatum	→	→	↗	→	Striatum	→	→	↗	→
Corpus callosum	↘	→	↗	→	Corpus callosum	→	→	↗	→
Internal capsule	—	—	—	→	Internal capsule	—	—	—	→

2 6 14 30 60 2 6 14 30 60

Potassium

Cortex	→	→	↗	→	Cortex	→	→	↗	→
Striatum	→	→	↗	→	Striatum	→	→	↗	→
Corpus callosum	→	↗	↗	→	Corpus callosum	→	↗	↗	→
Internal capsule	—	—	—	→	Internal capsule	—	—	—	→

2 6 14 30 60 2 6 14 30 60

Calcium

Cortex	→	→	→	→	Cortex	→	→	→	→
Striatum	→	→	↗	→	Striatum	→	→	→	→
Corpus callosum	→	→	→	→	Corpus callosum	→	→	→	→
Internal capsule	—	—	—	→	Internal capsule	—	—	—	→

2 6 14 30 60 2 6 14 30 60

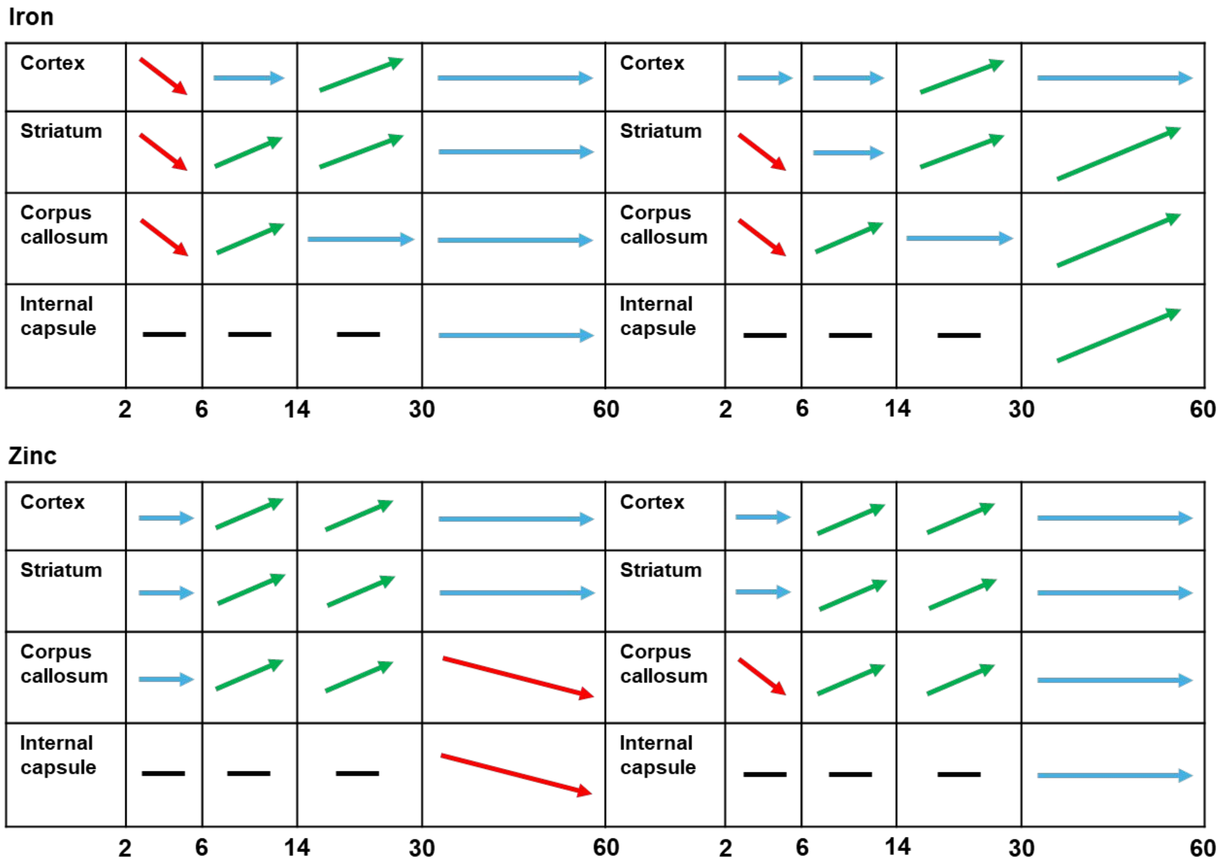

Figure S1. The dynamics of elemental changes (statistically significant differences in the elemental accumulation between the subsequent points of time) occurring in selected brain areas during postnatal development in the offspring of mothers fed during pregnancy with the ketogenic (K) or standard fodder (N). Statistically relevant increases of mass deposits of elements found for the examined periods were marked as the green sloping up arrows, whilst statistically relevant decreases as the sloping down red arrows. The blue arrow means no statistically significant differences between the subsequent points of time, and the black line indicates the lack of data enabling the performance of analysis for particular time period. The verification of the statistical significance of the observed differences was based on the Mann–Whitney *U* test (95% confidence level).
